# DuCLOX-2/5 Inhibition Attenuates Inflammatory Response and Induces Mitochondrial Apoptosis for Mammary Gland Chemoprevention

**DOI:** 10.3389/fphar.2018.00314

**Published:** 2018-04-06

**Authors:** Swetlana Gautam, Atul K. Rawat, Shreesh R. Sammi, Subhadeep Roy, Manjari Singh, Uma Devi, Rajnish K. Yadav, Lakhveer Singh, Jitendra K. Rawat, Mohd N. Ansari, Abdulaziz S. Saeedan, Dinesh Kumar, Rakesh Pandey, Gaurav Kaithwas

**Affiliations:** ^1^Department of Pharmaceutical Sciences, School of Biosciences and Biotechnology, Babasaheb Bhimrao Ambedkar University (A Central University), Lucknow, India; ^2^Center for Biomedical Research, Sanjay Gandhi Post Graduate Institute of Medical Sciences Campus, Lucknow, India; ^3^Department of Microbial Technology and Nematology, CSIR-Central Institute of Medicinal and Aromatic Plants, Lucknow, India; ^4^Department of Pharmaceutical Sciences, Faculty of Health and Medical Sciences, Sam Higginbottom Institute of Agricultural Sciences and Technology, Allahabad, India; ^5^Department of Pharmacology, College of Pharmacy, Prince Sattam Bin Abdulaziz University, Al-Kharj, Saudi Arabia

**Keywords:** DuCLOX-2/5 inhibition, angiogenesis, apoptosis, cyclooxygenase, lipoxygenase, NMR, Zaltoprofen, Zileuton

## Abstract

The present study is a pursuit to define implications of dual cyclooxygenase-2 (COX-2) and 5-lipoxygenase (5-LOX) (DuCLOX-2/5) inhibition on various aspects of cancer augmentation and chemoprevention. The monotherapy and combination therapy of zaltoprofen (COX-2 inhibitor) and zileuton (5-LOX inhibitor) were validated for their effect against methyl nitrosourea (MNU) induced mammary gland carcinoma in albino wistar rats. The combination therapy demarcated significant effect upon the cellular proliferation as evidenced through decreased in alveolar bud count and restoration of the histopathological architecture when compared to toxic control. DuCLOX-2/5 inhibition also upregulated levels of caspase-3 and caspase-8, and restored oxidative stress markers (GSH, TBARs, protein carbonyl, SOD and catalase). The immunoblotting and qRT-PCR studies revealed the participation of the mitochondrial mediated death apoptosis pathway along with favorable regulation of COX-2, 5-LOX. Aforementioned combination restored the metabolic changes to normal when scrutinized through ^1^H NMR studies. Henceforth, the DuCLOX-2/5 inhibition was recorded to import significant anticancer effects in comparison to either of the individual treatments.

## Introduction

Accruing numbers of factors involved in tumorigenesis, mounting evidence indicates that the inflammatory microenvironment accounts for the tumor development. Arachidonic acid (AA) and its metabolites involve the presumed convincing role in cancer biology (Hammamieh et al., 2007; Greene et al., 2011). Prolonged treatment with the non-steroidal anti-inflammatory drugs (NSAIDs) was well proved evidence to be associated with a lower risk of the several cancers, including mammary gland cancer (Dubois, 2000; Jänne and Mayer, 2000; Joyce and Pollard, 2009; Qian and Pollard, 2010).

AA is an essential fatty acid that plays a key role in metabolism; cell signaling and inflammation in mammals. The activation of the enzyme phospholipase A_2_ releases AA from cell membrane, which is further metabolized by the two key enzymes, cyclooxygenase (COX) and lipoxygenase (LOX) (Kawai et al., 2002). The metabolic end products of AA are termed as eicosanoids, which plays an important role in inflammation, development, reproductive process and inflammation-associated diseases including cancer. COX is an enzyme that metabolizes the AA into prostaglandins and is expressed in two isoforms. COX-1 is ubiquitously present in all cells, whereas COX-2 is only expressed in pathological conditions, for instance, in malignant and inflamed cells. COX-2 has been first noted to be up-regulated in colorectal cancer (EI-Hakim and Langdon, 1991; Eberhart et al., 1994; Gautam et al., 2016).

The interconnection between inflammation, cancer progression, COX-2 and 5-LOX products has invariably been of interest. Published reports have endorsed the over expression of COX and LOX in several cancers which depicts the link between cancer and inflammation. Emerging evidences proclaimed upsurge production of COX-2 and 5-LOX products in malignant cells (Harris, 2009; Harris et al., 2014). These enzymes interfere with normal physiological function within the cells and take part in the apoptosis, angiogenesis and invasiveness, proliferation and conversion of pro-carcinogen to carcinogens (Nie et al., 2000, 2001; Ye et al., 2005). While the expression of individual enzyme, the dual inhibition of AA metabolism has also been increasingly appreciated for their importance in cancer progression. Since no experimental data has been exercised in the direction of dual COX and LOX inhibition impeding to cancer, suggesting plethora of biochemical/physiological element needs to be understood and performed (Dempke et al., 2001; Ristimäki et al., 2002; Schneider and Pozzi, 2011). Authors considered it worth elaborating the effect of dual inhibition of AA metabolism on cancer progression. The proposed study presents the insight of differing roles of the enzymes COX-2 and 5-LOX in mammary gland carcinogenesis and emphasizes the potential of DuCLOX inhibition as target chemotherapy for cancer.

## Materials and methods

### Drug and chemicals

Zaltoprofen was procured from the local market under the brand-name zaltokin from IPCA laboratory's Ltd., India; and zileuton was solicited as API from Shanghai worldyang chemical co. Ltd., China. All other chemicals were of analytical grade and procured from Genetix Biotech Asia Pvt. Ltd. New Delhi, India else otherwise stated within the text.

### Animals

Wistar strain of female albino rats of (100–120 g) were procured from the central animal house. Animals were housed under standard condition (23°C, 12 h light/dark cycle), with a free access to a standard pellet diet and water *ad libitum*. Animals were acclimatized for a period of 2 weeks prior to the commencement of the experiment, and the study was performed according to the standard ethical guidelines and approved by the Institutional Animal Ethics Committee (BBDNIIT/IAEC/020/2014).

### Experimental design

Animals were randomized and divided into 5 groups of 6 animals each. Group I (control 0.9% normal saline, 3 ml/kg, p.o.); Group II (toxic control, MNU 47 mg/kg, i.v.); Group III (Zaltoprofen, 10 mg/kg, p.o.); Group IV (Zileuton, 10 mg/kg, p.o.); and Group V (Zaltoprofen, 5 mg/kg, p.o + Zileuton, 5 mg/kg, p.o.). Mammary gland carcinoma was induced by single i.v. injection of MNU on 7th day after commencing the treatment. The animals were recorded for the autonomic control though electrocardiogram (ECG) and Heart rate variability (HRV) paradigms on 119th day. The animals were sacrificed by using light ether anesthesia on 120th day and mammary gland tissue was collected. The whole mount tissue was assessed for their morphological changes using carmine staining, and the rest was further evaluated for other parameters.

### Antioxidant markers

The mammary gland tissues (10% w/v) were homogenized in 0.15 M KCl and centrifuged at 10,000 rpm. The supernatants were scrutinized for biochemical parameters, including thiobarbituric acid reactive substances (TBARs), superoxide dismutase (SOD), catalase, protein carbonyl (PC) and glutathione (GSH) using the methods established at our laboratory (Kaithwas et al., 2007, 2011; Kaithwas and Majumdar, 2012).

### Hemodynamic studies

Animals were anesthetized using ketamine hydrochloride (100 mg/kg, i.m.) and diazepam (5 mg/kg, i.m.) in combination and mounted on a wax tray. The ECG signals were recorded using platinum hook electrodes connected to bio amplifier (ML-136) and single channel PowerLab (ML-826) (AD Instruments, Australia). The ECG signals were used to perform HRV analysis (Labchard PRO-8, AD Instruments, Australia) using the method described previously (Roy et al., 2018).

### Carmine staining of whole mount mammary gland

Whole mount of the mammary gland was prepared using Carnoy's fixative solution using the methods previously established at our laboratory. The slides were examined under the microscope to assess the number of terminal end buds (TEBs), alveolar buds (ABs) type 1 and 2, and differentiation (DF) score. Detailed procedure for the same has been described by us previously (Manral et al., 2016; Rani et al., 2016).

### Histopathology of mammary gland tissue

Mammary gland tissues were appraised histopathologically using haematoxyline and eosin staining (H&E). 5 μm sections of mammary gland tissue were prepared and stained with H&E using the procedure explained elsewhere (Belur et al., 1990; Murray et al., 2009; Rani et al., 2018).

### Evaluation of caspase-3 and caspase-8

The serum samples were assayed for the caspase-3 and caspase-8 levels using DEVD-AFC complex and IETD-AFC complex principles respectively, following the instruction manual (Bustamante et al., 2002; Martinez et al., 2004; Gautam et al., 2018).

### Western blotting

Protein samples were prepared from the mammary gland tissue through acetone precipitation and quantified by using the Bradford reagent (Ahmad and Sharma, 2009). SDS-PAGE analysis was performed following the principles of Laemmli with slight modifications (Laemmli, 1970). Briefly, protein samples were mixed with sample buffer (125 mM Tris-HCl, pH 6.8, 20% glycerol, 4% SDS, 0.05% bromophenol blue, 10% 2-mercaptoethanol). A 30 μg of protein sample was allowed to resolve through 12% polyacrylamide gel using SDS-PAGE (GX-SCZ2+, Genetix Biotech Asia Pvt. Ltd. New Delhi). The proteins as resolved through SDS-PAGE were transferred to a PVDF membrane (IPVH 00010 Millipore, Bedford, MA USA) using semidry transfer (GX-ZY3), Genetix Biotech Asia Pvt. Ltd., New Delhi. Subsequently, membrane was blocked with 3% BSA and 3% non-fat milk in TBST for 2 h and incubated overnight with primary antibody against B cell lymphoma-2 (BCL-2) (SC-7382), B cell lymphoma-xl (BCL-XL) (MA-5-15142), BAX (SC-23959), BAD (SC-8044), Voltage dependent anion channels (VDAC) (390996), Apoptotic protease activating factor-1 (APAF-1) (SC-65891), Procaspase-9 (SC-73548), COX-2 (MA5-14568), 5-LOX (PA1-16953), and β-actin (MA5-15739-HRP) (Pierce, Thermo scientific) (1:3000 dilution). The membrane was washed with TBST thrice and incubated with HRP conjugated rat antimouse secondary antibody (31430, 1:5000 dilutions) (Pierce Thermo Scientific) at room temperature for 2 h. The signals were detected using an enhanced chemiluminescence substrate (Western Bright ECL HRP substrate, Advansta, Melanopark, California, US). The Quantification of protein was done through densiometeric digital analysis of protein bands using ImageJ software (Laemmli, 1970; Towbin et al., 1979).

### Quantitative RT-PCR

Primers for real time were designed online using the primer quest tool from the IDT DNA technologies' website (www.idtdna.com). The amplicon size was kept between 100 and 200 base pairs, GC% was kept above 50% and melting temperature was kept between 58 and 62°C. The specific sequences of the forward and reverse primers are specified in Table 1.

**Table 1:** Sequence of forward and reverse primers used for quantitative RT-PCR.

**Primer**	**Sequence**
*Bcl-2* F	GTG GAT GAC TGA GTA CCT GAA
*Bcl-2* R	GAG ACA GCC AGG AGA AAT CAA
*Bcl-xl* F	CCC TCG TAT CTG GAA GCC AC
*Bcl-xl* R	CAG CGG AGA CCT CGT TTT CT
*Bad* F	CTC CGA AGA ATG AGC GAT GAA
*Bad* R	ATC CCA CCA GGA CTG GAT AA
*Bax* F	TGC TAC AGG GTT TCA TCC AG
*Bax R*	GAC ACT CGC TCA GCT TCT T
*Apaf-1* F	GAA CAT AGA CTC CCG GGT AAA G
*Apaf-1* R	CTT GTC TCC CAG ACC CTT ATT G
*Cas- 9* F	GGC TCT CTG GCT TCA TTC TT
*Cas- 9* R	GGG TCC AGC TTC ACT ACT TTC
*Vdac* F	GGA GTT TGG TGG CTC CAT TTA
*Vdac* R	GAC CTG ATA CTT GGC TGC TAT TC
*Cyto-c* F	TCC ATT TCC CTT CCT TGG GC
*Cyto-c* R	ATC GGG GCT GTC CAA CAA AA
*COX-2* F	CCT TCG GGC ACA TGG TAA GT
*COX-2* R	CAG CCC ACT CCA TAC TGC AA
*5-LOX* F	CTA CAA GTA CTC CGA CGA CA
*5-LOX* R	AAG TAA CCG GTG CCA TAT CC

Total RNA was extracted from mammary gland tissue using trizol reagent (Invitrogen, Life Technologies) according to the manufacturer‘s instructions. Briefly, tissues were washed off treatment plates using 0.1% DEPC water. The tissues were crushed in 250 μl trizol reagent using micro pestles. Another 750 μl of trizol reagent was added to make the final volume to 1 ml, followed by addition of 200 μl of chloroform and mixing for 2–5 min on a vortex mixer. The suspension was then centrifuged at 14,000 rpm, 4°C for 15 min and upper aqueous phase was gently pipette out in the fresh vials. RNA was precipitated by addition of 500 μl chilled isopropanol. The vials were kept at room temperature for 10 min and were centrifuged at 14,000 rpm, 4°C for 10 min and RNA pellet so obtained was washed twice with 75% ethanol (chilled) at 7,500 rpm, 4°C for 5 min. The RNA pellet was finally dissolved in 15 μl of 0.1% DEPC water. To quantify RNA absorbance was read using nano drop (Qua Well Q5000). cDNA synthesis was done from 1 μg of total mammary gland RNA in a 96 well thermal cycler (BioRad, C1000) with steps including, incubation at 25°C for 10 min, 37°C for 120 min, 85°C for 5 min and 4°C forever RNA using high capacity cDNA synthesis Kit (Applied Biosystems). cDNA sample were quantified using nanodrop and were stored at −80°C until use. 125 ng of cDNA was used as a template for each reaction of qRT-PCR with β-actin as housekeeping control using light cycler 480 machine (Roche Diagnostics, Germany). For each primer pair, a melting curve analysis was performed according to instrument. The program in brief was an initial incubation of 50°C for 2 min hold (UDG incubation) and 95°C for 10 min followed by 40 cycles at 95°C for 15 s (denaturation), 58°C for 30 s (annealing) and final extension at 72°C for 20 s. Differential expression was calculated by 2−ΔΔCT method. β-actin was used as internal control and used to normalize ratios between samples (Giulietti et al., 2001; Kaithwas et al., 2007; Roy et al., 2017).

### NMR based serum metabolomics analysis

For NMR based metabolomics analysis, the serum samples stored at −80°C were thawed, vortexed, and centrifuged at 10,000 rpm on room temperature. Next, the serum samples were analyzed by high resolution 1D 1H NMR spectroscopy on a Bruker AVANCE III 800 MHz NMR spectrometer equipped with TXI Cryoprobe. For small metabolite profiling, the standard 1D ^1^H Carr-Purcell-Meiboom-Gill (CPMG) NMR spectra was recorded on all serum samples after processing the sera samples as described previously (Rawat et al., 2016a,b). The NMR spectra obtained from different study groups was afterwards imported into the Topspin AMIX software (Bruker GmbH, Rheinstetten, Germany) and segmented into 0.02-ppm bins (buckets) in the spectral region 0.7–8.5 ppm, excluding the residual water signal ranging from (4.65–5.01) ppm. The CPMG data matrix was Pareto scaled for each NMR variable (i.e., for each bin) and subsequently subjected to multivariate/univariate analysis using MetaboAnalyst 3.0 (Xia et al., 2009, 2015). First, we performed the multivariate analysis based on standard algorithms–PCA (principal component analysis), PLS-DA (partial least-squares discriminant analysis), and OPLS-DA (Orthogonal Projection to Latent Structure with Discriminant Analysis)-to evaluate the metabolic differences between the control and treated groups. The unsupervised PCA was performed initially to get an overview of the grouping trends and to separate the effective treatment dose. Next, we performed the supervised PLS-DA modeling (pair wise as well as combined) to discriminate the groups based on metabolic differences. The metabolites responsible for group discrimination were evaluated based on the variable importance on projection scores (i.e., VIPs) >1. The goodness-of-fit parameters R^2^ and Q^2^, which relate to the explained and predicted variance, respectively, were used to evaluate the PLS-DA model performance. Further, univariate analysis was applied to assess the significance for the change in the metabolic profile for the pair wise and combined analysis (*t*-test and ANOVA respectively), *p* < < 0.05 was used as the criterion for statistical significance (Wishart et al., 2007; Guleria et al., 2014; Kumar et al., 2016).

### Statistical analysis

All data were presented as mean ± SD and analyzed by one-way ANOVA followed by Bonferroni test and for the possible significance identification between the various groups. c/^*^p < 0.05, b/^**^p < 0.01, and a/^***^p < 0.001 were considered as statistically significant. Statistical analysis was performed using Graph Pad Prism software (5.02).

## Results

### Change in oxidative stress markers in MNU-induced mammary gland carcinogenesis

There was an upsurge (0.23 ± 0.07 nM of MDA/μg of protein) in TBAR's level after MNU administration in comparison with normal control (0.09 ± 0.02) rats. A significant dose dependent decrease in the TBAR's level was observed subsequent to zaltoprofen (0.15 ± 0.05), zileuton (0.09 ± 0.01) and in combination treatment (0.08 ± 0.00). Decrease in the enzymatic activity of SOD (0.22 ± 0.02 units of SOD/mg of protein) and catalase (1.51 ± 0.06 nM of H_2_O_2_/min/mg of protein) was scrutinized in the animals treated with MNU. Combination treatment helped to restore the levels of SOD and catalase more efficiently in comparison to test drugs alone. Similar patterns of results were scrutinized for the GSH (Table 2).

**Table 2:** Effect of Zaltoprofen and Zileuton on oxidative stress markers against MNU induced mammary gland carcinoma.

**Groups**	**TBARS (nM of MDA/mg of protein)**	GSH^*^10^−4^ (mg %)	**SOD (unit of SOD/mg of protein)**	**Catalase (nM of H_2_O_2_/min/mg of protein)**	**Protein Carbonyl (nM/ml)**
Group I Control (Normal Saline, 3 ml/kg, i.p.)	0.088 ± 0.02	0.076 ± 0.00	0.256 ± 0.02	1.66 ± 0.09	55.98 ± 13.06
Group II Toxic Control (MNU 47 mg/kg, i.v.)	0.229 ± 0.07^***^	0.065 ± 0.00	0.224 ± 0.02	1.51 ± 0.06	78.10 ± 9.04
Group III Zaltoprofen (10 mg/kg, p.o.)	0.150 ± 0.05^a^	0.061 ± 0.00	0.205 ± 0.01^a^	1.28 ± 0.01^***^	72.87 ± 5.47
Group IV Zileuton (10 mg/kg, p.o.)	0.089 ± 0.01^a^	0.077 ± 0.01	0.305 ± 0.00^b^	1.53 ± 0.15	66.59 ± 31.52
Group V Zaltoprofen+ Zileuton (5 + 5 mg/kg, p.o.)	0.086 ± 0.00^***^	0.066 ± 0.00	0.607 ± 0.29^***^	1.32 ± 0.13^***^	62.04 ± 3.05

(Values are Mean ± SD), each group contains eight animals. Comparisons were made on the basis of the one-way Anova followed by Bonferroni test. All groups were compared to the MNU treated group (

*** p < 0.001). Group V were compared to the III and IV treated group (

a *p < 0.001*,

b *p < 0.01)*.

### Effects of zaltoprofen and zileuton on hemodynamic changes

**Figure 1:**
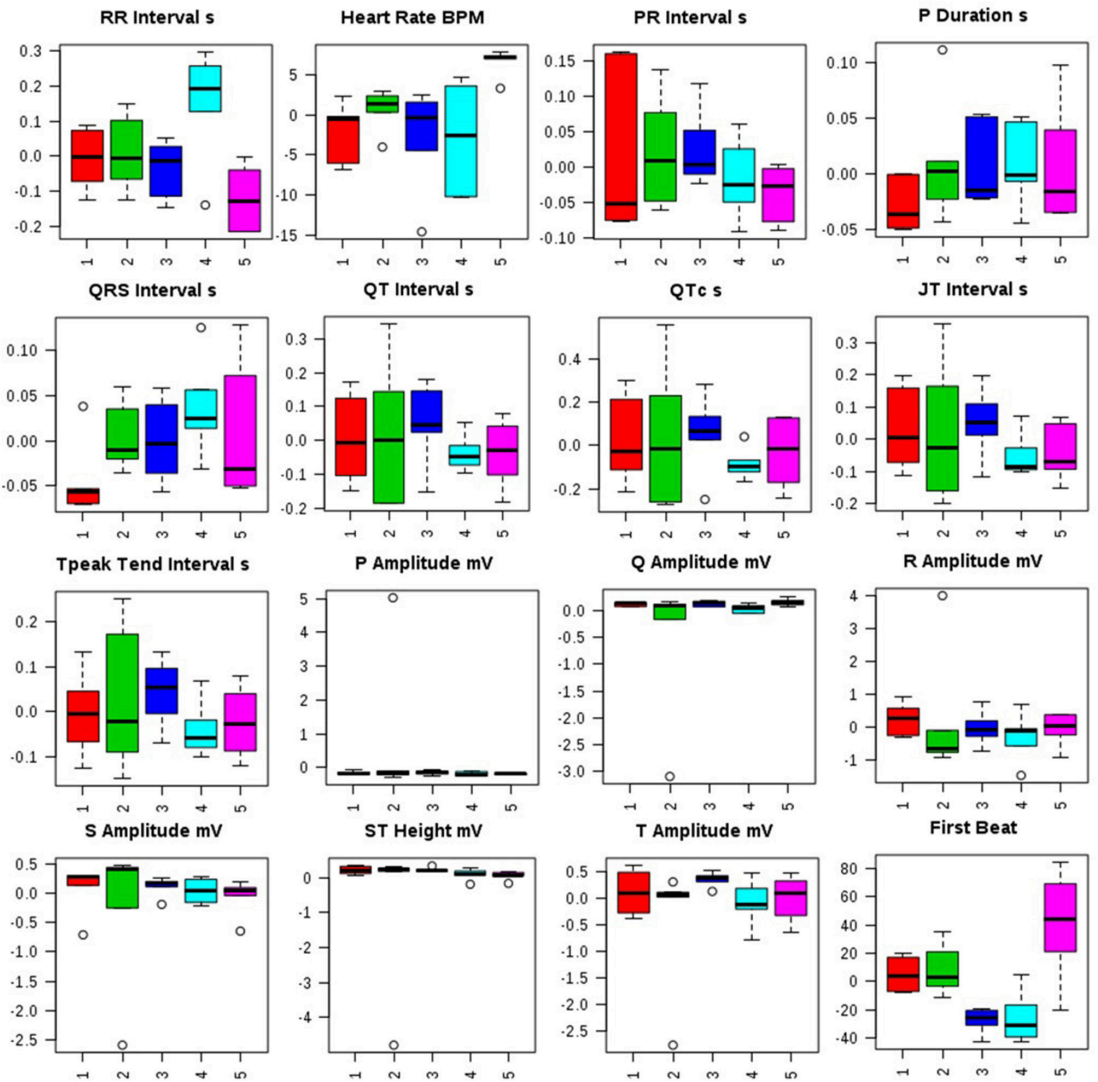
Effects of Zaltoprofen and Zileuton treatment on ECG recording. Representative box-cum-whisker plots showing quantitative variations of relative signal integrals for autonomic dysfunction relevant in the context of pathophysiology of mammary gland cancer. Groups were differentiated as: 1-Control (Normal saline, 3 ml/kg, p.o.), 2-Toxic control (MNU 47 mg/kg, i.v.), 3- Zaltoprofen (10 mg/kg, p.o. + MNU 47 mg/kg, i.v.), 4- Zileuton (10 mg/kg, p.o. + MNU 47 mg/kg, i.v.) and 5-Zaltoprofen+Zileuton-(5 +5 mg/kg, p.o. + MNU 47 mg/kg, i.v.). For presented ECG recordings, the VIP score >1 and statistical significance is at the level of *p* ≤ 0.05. In the box plots, the boxes denote interquartile ranges, horizontal line inside the box denote the median, and bottom and top boundaries of boxes are 25 and 75th percentiles, respectively. Lower and upper whiskers are 5 and 95th percentiles, respectively.

Administration of MNU demonstrated aberration in the ECG profile characterized by QT prolongation; QRS prolongation; marginal decrease in HR; increased dispersion of P wave amplitude in comparison to control (Figure 1, Figure S1).

**Figure 2:**
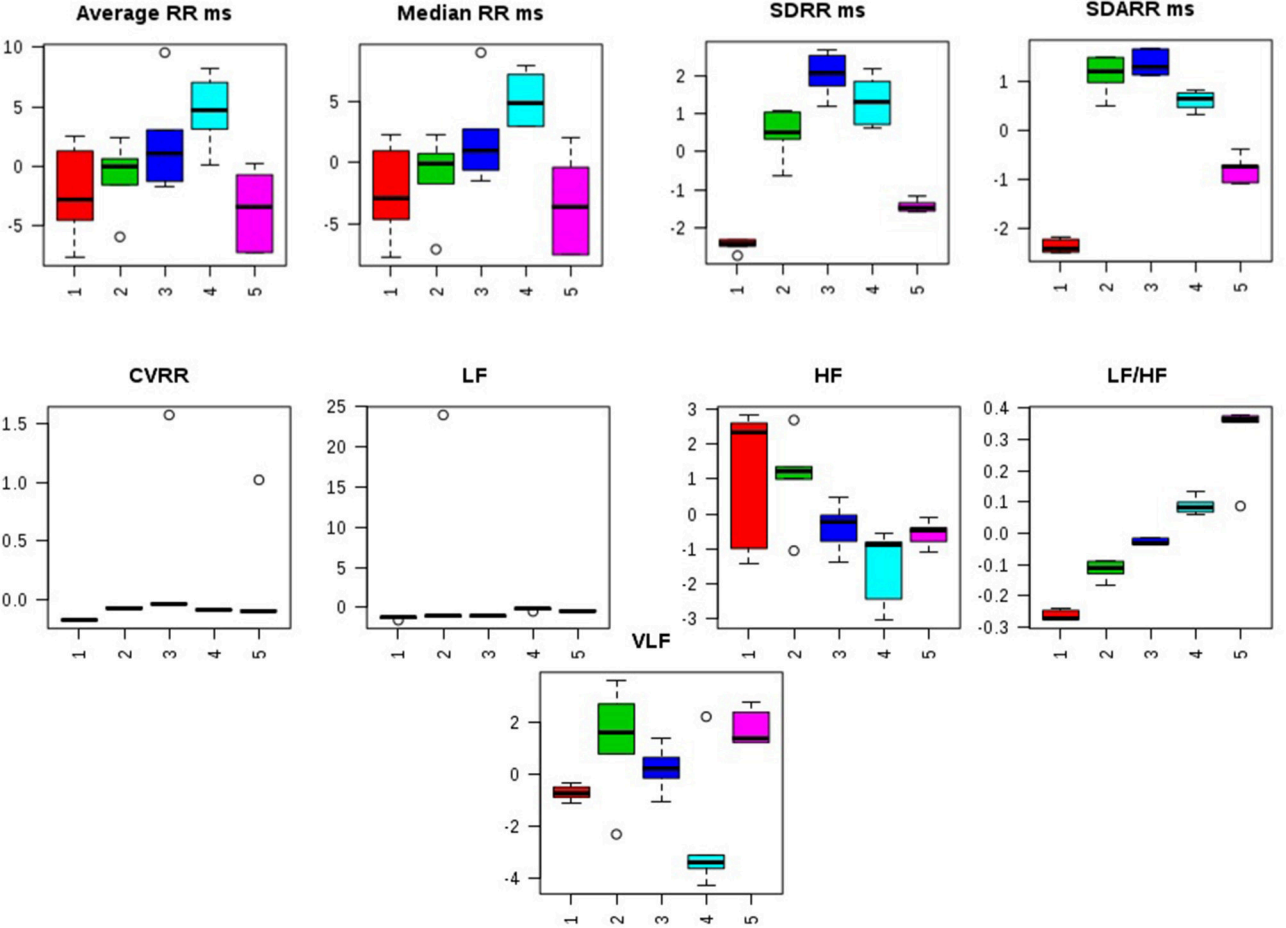
Effects of Zaltoprofen and Zileuton treatment on HRV. Representative box-cum-whisker plots showing quantitative variations of relative signal integrals for HRV parameters relevant in the context of pathophysiology of mammary gland cancer. Groups were differentiated as: 1- Control (Normal saline, 3 ml/kg, p.o.), 2-Toxic control (MNU 47 mg/kg, i.v.), 3- Zaltoprofen (10 mg/kg, p.o. + MNU 47 mg/kg, i.v.), 4- Zileuton (10 mg/kg, p.o. + MNU 47 mg/kg, i.v.) and 5-Zaltoprofen + Zileuton-(5 + 5 mg/kg, p.o. + MNU 47 mg/kg, i.v.). For presented heart rate variablity, the VIP score >1 and statistical significance is at the level of *p* ≤ 0.05. In the box plots, the boxes denote interquartile ranges, horizontal line inside the box denote the median, and bottom and top boundaries of boxes are 25 and 75th percentiles, respectively. Lower and upper whiskers are 5 and 95th percentiles, respectively.

Distorted HRV profile was recorded for the time domain (Average RR, Median RR, SDRR, SDARR and CVRR) and frequency domain (LF, HF, and LF/HF) parameters after MNU treatment. Treatment with zaltoprofen and zileuton exerted favorable effects toward restoring the HRV paradigms toward normal with more profound effects by combination regime (Figure 2, Figure S2).

### Carmine staining of whole mount's mammary gland

**Figure 3:**
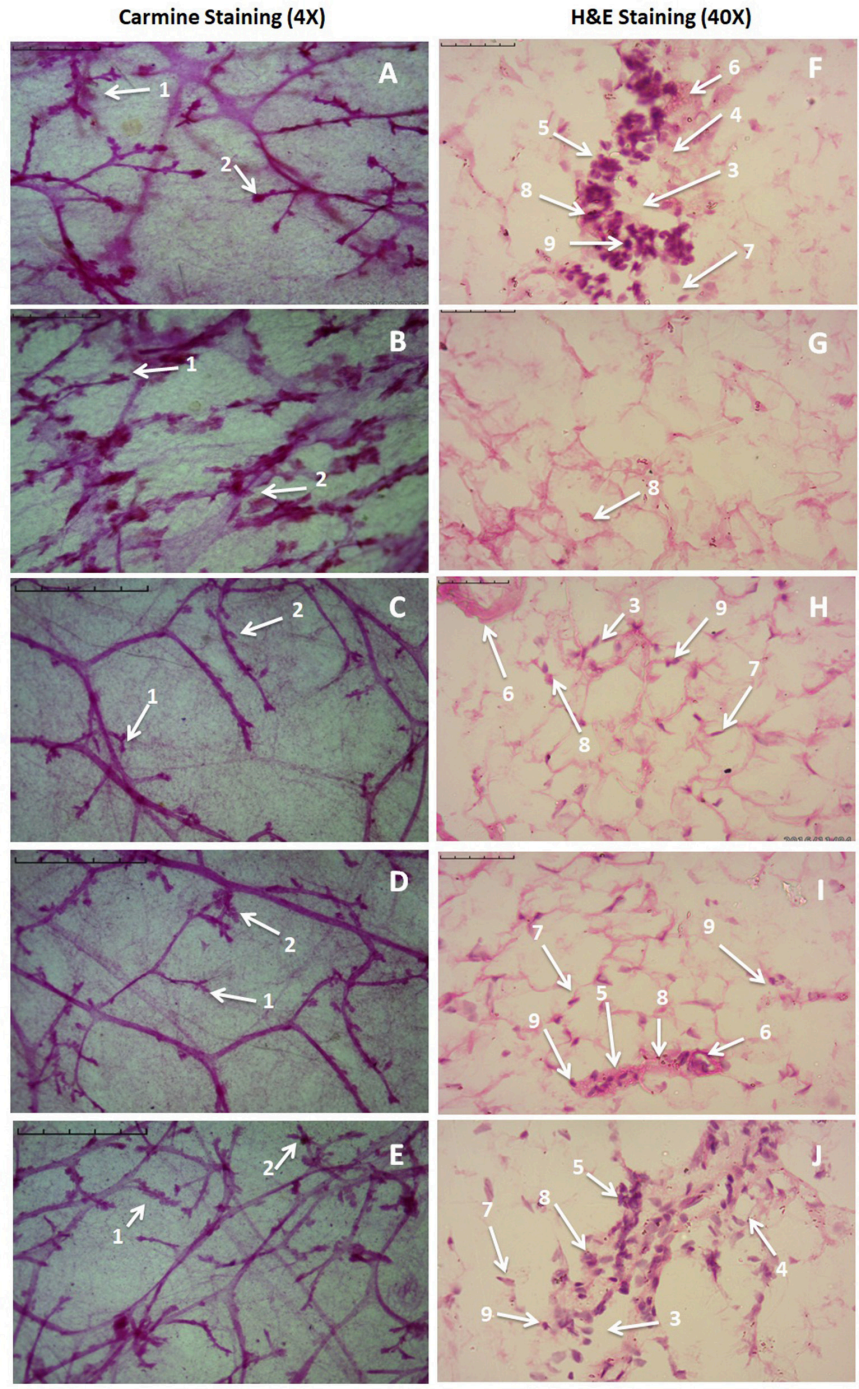
Microscopic evaluation of mammary gland tissue of the animal treated with Zaltoprofen, Zileuton and their combination through carmine and H&E staining. Whole mount carmine alum staining of ductal epithelium reveals the presence of lobules (1) and AB (2) **(A–E)**. The extent of AB and lobules formation was excessive in the MNU treated group **(B)** which was subsided through respective treatment zaltoprofen, zileuton and a combination **(C–E)**. The images were captured under microscope with 4 X magnification. H&E staining of respective groups **(F–J)** revealed duct (3), adipocytes (4), LCT (5), DCT (6), MEC (7), lymphocytes (8) and CEC (9) in control **(F)** as well as treated groups zaltoprofen, zileuton and a combination treatment respectively **(H–J)**. In MNU treated group **(G)**, the cell morphology was distorted and cell organelles were absent. The images were captured under microscope with 40 X magnification.

Zaltoprofen and zileuton treatment, and in combination therapy (Figures 3C–E), offered significant protection similar to control group (Figure 3A) against MNU induced degeneration of cellular morphology (Figure 3B). MNU treatment was marked increase in the lobules and AB count, representing cellular proliferation, and combination therapy afforded a marked protection against the same (Table 3).

**Table 3:** Effect of Zaltoprofen and Zileuton on differentiation of mammary gland against MNU induced mammary gland carcinoma.

**Groups**	**AB1**	**AB2**	**AB1 +AB2**	**Lobules**	**DF.SCORE 1 (AB1+AB2+ Lobules)**	**DF.SCORE 2 (Lobules/AB1+AB2)**
Control (NS, 3 ml/kg, i.p.)	14.5 ± 0.70	0.5 ± 0.70	15 ± 1.41	1 ± 0.00	16 ± 1.41	0.66 ± 0.00
MNU (47 mg/kg, i.v.)	21.5 ± 6.36	11 ± 2.8	32.5 ± 9.19	6.5 ± 2.12	39 ± 11.31	0.19 ± 0.00
Zaltoprofen (10 mg/kg, p.o.)	15.5 ± 3.53	8 ± 0.00	23.5 ± 3.53	1 ± 1.41^***^^c^	27.5 ± 2.12^**^	0.17 ± 0.08^**^^a^
Zileuton (10 mg/kg, p.o.)	17 ± 2.82	8.5 ± 0.70	25.5 ± 2.12	5 ± 2.82	30.5 ± 0.70	0.20 ± 0.12^**^
Zaltoprofen+Zileuton (5 + 5 mg/kg, p.o.)	14 ± 2.82^*^	6.5 ± 3.53^**^	20.5 ± 6.36^**^	4.5 ± 2.12	25 ± 8.48^**^	0.21 ± 0.03^*^

(Values are Mean ± SD), each group contains eight animals. Comparisons were made on the basis of the one-way Anova followed by Bonferroni test. All groups were compared to the MNU treated group (

* *p < 0.05*,

** *p < 0.01*,

*** p < 0.001). Group V were compared to the III and IV treated group (

a *p < 0.001*,

c *p < 0.05)*.

### Effects of zaltoprofen and zileuton on mammary gland morphology

H&E staining of the mammary gland tissue revealed the presence of duct, lymphocytes, adipocytes, loose connective tissue (LCT), dense connective tissue (DCT), cuboidal epithelial cells (CEC), and myoepithelial cells (MEC) in case of control animal (Figure 3F). MNU treated groups were observed for the loss of LCT, DCT and adipocytes, and scattered cuboidal epithelial cells (Figure 3G). Treatment with zaltoprofen and zileuton restored the cellular architecture as evident through presence of lymphocytes, adipocytes, LCT, DCT, and MEC (Figures 3H,I). It would be appropriate to remark that the combination therapy embarked a more profound effect in comparison to monotherapy (Figure 3J).

### Effects on caspase-3 and -8

**Figure 4:**
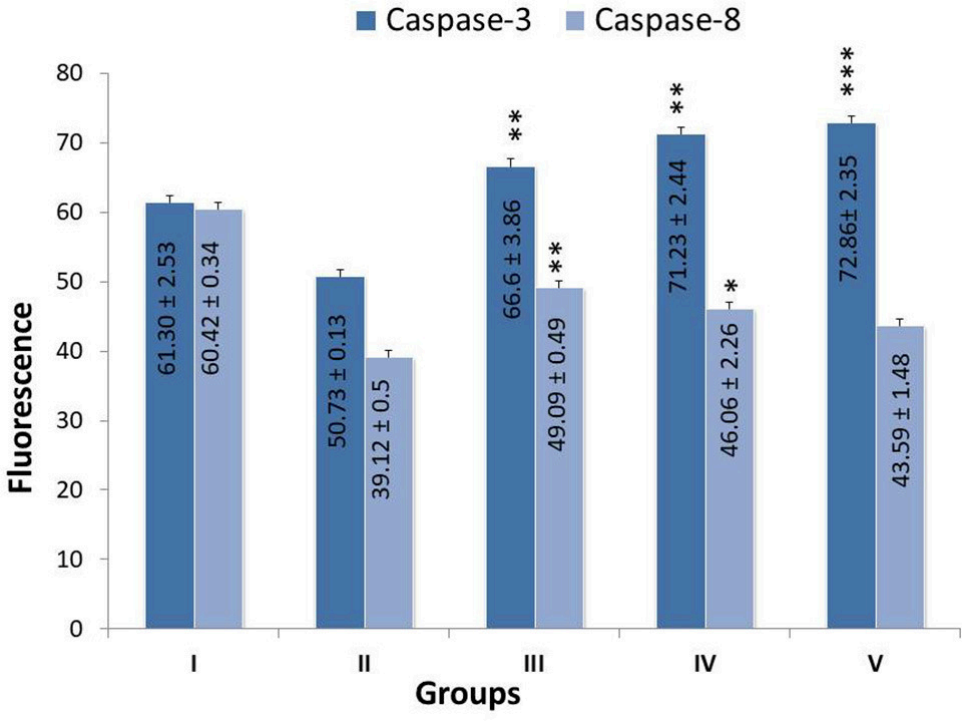
Effect of Zaltoprofen, Zileuton and their combination on caspase3 and caspase8. The activity of caspase was detected by commercial fluorescence based assay in Group I-Control (Normal saline, 3 ml/kg, p.o.), II- Toxic control (MNU 47 mg/kg, i.v.), III- Zaltoprofen (10 mg/kg, p.o. + MNU 47 mg/kg, i.v.), IV- Zileuton (10 mg/kg, p.o. + MNU 47 mg/kg, i.v.) and V- Zaltoprofen + Zileuton (5 + 5 mg/kg, p.o. + MNU 47 mg/kg, i.v.). Data are expressed as mean+ SD of individual groups. Comparisons were made by the one-way ANOVA followed by Bonferroni multiple test. All groups were compared to the MNU treated group (^*^p < 0.05, ^**^p < 0.01, ^***^p < 0.001).

Treatment with monotherapy and combination therapy of zaltoprofen and zileuton upregulated the apoptotic markers caspase-3 and caspase-8, when compared with MNU treatment (Figure 4).

### Western blot

**Figure 5:**
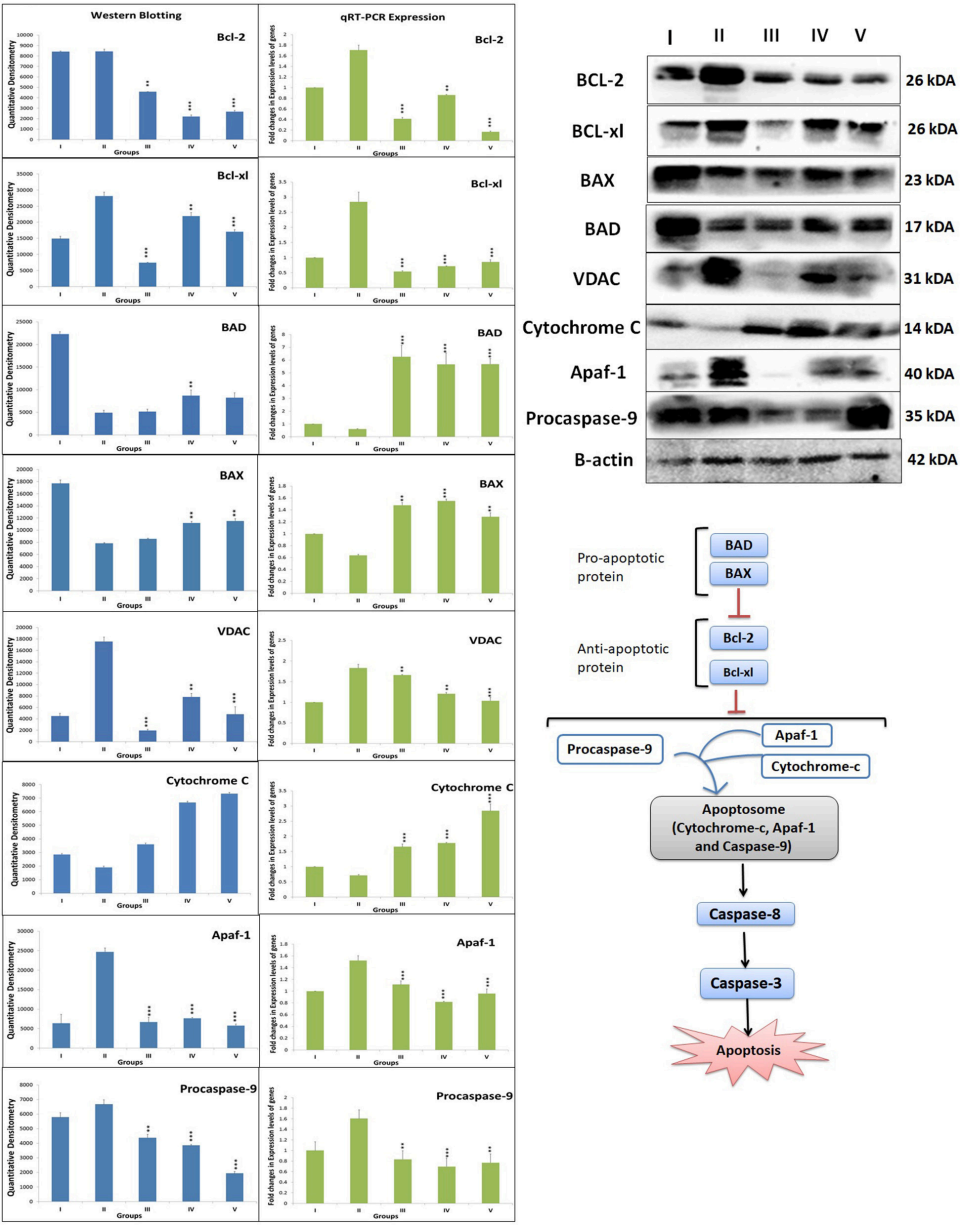
DuCLOX-2/5 mediated activation of mitochondrial associated protein signaling in mammary gland cells. Protein extracted from individual groups [I-Control (Normal saline, 3 ml/kg, p.o.), II- Toxic control (MNU 47 mg/kg, i.v.), III- Zaltoprofen (10 mg/kg, p.o. + MNU 47 mg/kg, i.v.), IV- Zileuton (10 mg/kg, p.o. + MNU 47 mg/kg, i.v.) and V- Zaltoprofen + Zileuton (5 + 5 mg/kg, p.o. + MNU 47 mg/kg, i.v.)] were subjected to immunoblotting of proapoptotic (BAX and BAD) and anti-apoptotic (Bcl-2 and Bcl-xl) protein with downstream apoptotic markers (VDAC, cytochrome-c, Apaf-1 and procaspase-9) of respective pathway. mRNA expression of above mentioned protein were also in line with the findings of immunoblotting assay. β-actin was used as loading control. Each experiment was performed in triplicate. Values are presented as Mean ± SD. Comparisons were made by the one-way ANOVA followed by Bonferroni multiple test. All groups were compared to the MNU treated group (^*^p < 0.05, ^**^p < 0.01, ^***^p < 0.001).

When ascertained on the grounds of protein expression of mitochondrial apoptotic pathway, MNU administration was recorded for the upregulated expression of BCL-2, BCL-xl, VDAC, Apaf-1 and procaspase-9, and downregulation of BAD, BAX, and cytochrome-c. Zaltoprofen and zileuton, alone and in combination, demonstrated significant restoration of the apoptotic markers (Figure 5).

**Figure 6:**
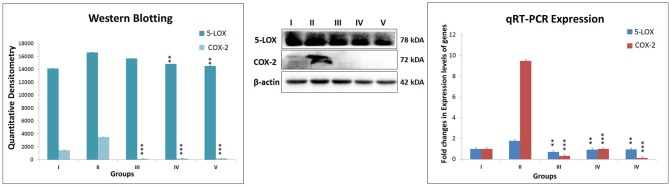
Expression level of protein of COX-2 and 5-LOX through western blot and levels of gene contributor through quantitative RT-PCR. Immunoblotting of respective individual group [I-Control (Normal saline, 3 ml/kg, p.o.), II- Toxic control (MNU 47 mg/kg, i.v.), III- Zaltoprofen (10 mg/kg, p.o. + MNU 47 mg/kg, i.v.), IV- Zileuton (10 mg/kg, p.o. + MNU 47 mg/kg, i.v.) and V- Zaltoprofen + Zileuton (5 + 5 mg/kg, p.o. + MNU 47 mg/kg, i.v.)] for COX-2 and 5-LOX. Excised mammary gland tissue sample lysed in trizol for RNA extraction and analyzed for the mRNA expression of COX-2 and 5-LOX by qRT-PCR. β-actin was used as loading control. Each experiment was performed in triplicate. Values are presented as Mean ± SD. Comparisons were made by the one-way ANOVA followed by Bonferroni multiple test. All groups were compared to the MNU treated group (^**^p < 0.01, ^***^p < 0.001).

While ascertaining the expression of COX-2 and 5-LOX proteins in the inflammatory pathway, the same were found to be overexpressed in MNU treated animals. Test drug's treatment, in particular, combination regime, modulated the expression for COX and LOX proteins favorably (Figure 6).

### Quantitative RT-PCR

After ascertaining that Zaltoprofen and zileuton, alone and in combination, treatment was leading to elevated death protein and inflammatory markers, our next objective was to identify the genomic contributors for the observed phenotype, which was achieved through quantitative PCR for genes BCL-2, BCL-xl, BAD, BAX, VDAC, Apaf-1, procaspase-9, cytochrome-c, COX-2, and 5-LOX. In comparison to the toxic, we observed a significant downregulated expression of gene BCL-2 (0.41, 0.86, and 0.16), BCL-xl (0.51, 0.71, and 0.85) and upregulation of BAD (6.26, 5.67, and 5.68), BAX (1.47, 1.55, and 1.28) in treatment groups of zaltoprofen, zileuton and combination treatment respectively. The fold change in relative expression of the genes of the cell death pathways, i.e., VDAC, Apaf-1, procaspase-9, cytochrome-c, and COX-2 and 5-LOX of the inflammatory pathway was, however, close to normal (Figure 5).

### ^1^H-NMR method for serum metabolites profiling

**Figure 7:**
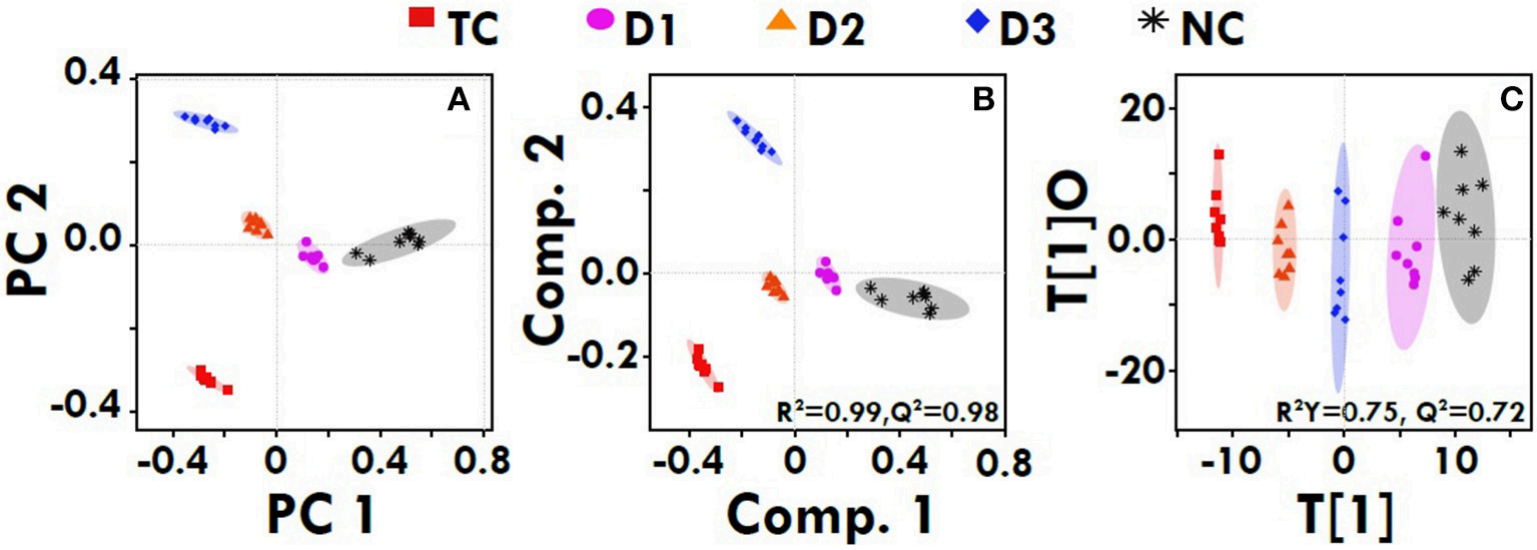
Multivariate analysis. The combined 2D PCA **(A)** and 2D PLS-DA **(B)** 2D OPLS-DA **(C)** score plots derived from cumulative analysis of 1D ^1^H CPMG NMR spectra comprising of all the groups: NC- Normal control (Normal saline, 3 ml/kg, p.o.), TC-Toxic control (MNU 47 mg/kg, i.v.), D1- Zaltoprofen (10 mg/kg, p.o. + MNU 47 mg/kg, i.v.), D2- Zileuton (10 mg/kg, p.o. + MNU 47 mg/kg, i.v.) and D3-Zaltoprofen+Zileuton-(5 + 5 mg/kg, p.o. + MNU 47 mg/kg, i.v.). Color circles indicate the 95% confidence interval for each class (9B). Color circles indicate the 95% confidence interval for each class.

The quantitative NMR profiles of sera were subjected to multivariate discriminaotry analysis to screen the different metabolites between the controls and tumor-bearing rat. The combined PCA score plot showed a clear pattern of clustering in different groups, and no outlier sample was detected (Figure 7A). As evident from Figure 7A, the PCA itself was able to give an excellent separation between the groups, where the treated group with dose zaltoprofen found to be closer to the normal control (NC) group compared to those treated with zileuton or combination. The supervised PLS-DA was used as a discriminatory model to distinguish between the groups and to identify the marker metabolites that differentiate the groups. The combined PLS-DA score plot for all the groups (Figure 7B) showed that the samples in various groups are well clustered; Toxic control (TC) being the farthest and zaltoprofen group being the closest to NC group. Additionally, OPLS-DA was also employed for combined analysis of spectral data which further support for the trends as observed in PCA and PLS-DA (Figure 7C). Similarly, the pairwise PCA, PLS-DA and OPLS-DA analyses were also performed comparing all the study groups toxic control, zaltoprofen, zileuton and combination therapy, with respect to the NC (Figure S3). Each pairwise model revealed that there are significant metabolic differences in treated rats compared to TC rats as evident from the model cross-validation parameters R^2^ and Q^2^, representing the explained variance and predictive capability of the model, respectively.

**Figure 8:**
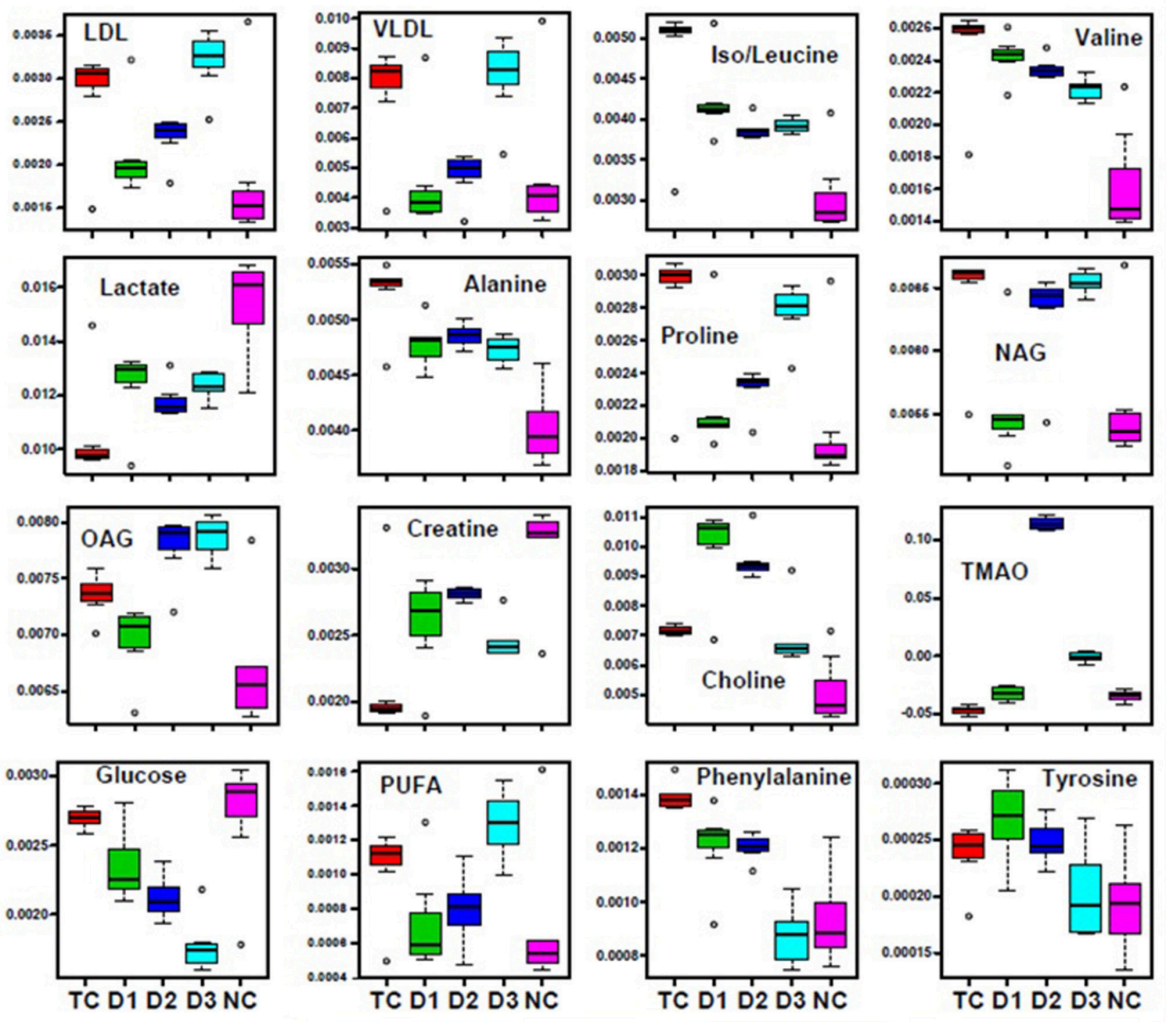
Biochemical effects of Zaltoprofen and Zileuton treatment. Representative box-cum-whisker plots showing quantitative variations of relative signal integrals for serum metabolites relevant in the context of pathophysiology of mammary gland cancer. Groups were differentiated as: NC- Normal control (Normal saline, 3 ml/kg, p.o.), TC-Toxic control (MNU 47 mg/kg, i.v.), D1- Zaltoprofen (10 mg/kg, p.o. + MNU 47 mg/kg, i.v.), D2- Zileuton (10 mg/kg, p.o. + MNU 47 mg/kg, i.v.) and D3-Zaltoprofen+Zileuton-(5 + 5 mg/kg, p.o. + MNU 47 mg/kg, i.v.). For presented metabolite entities, the VIP score >1 and statistical significance is at the level of *p* ≤ 0.05. In the box plots, the boxes denote interquartile ranges, horizontal line inside the box denote the median, and bottom and top boundaries of boxes are 25 and 75th percentiles, respectively. Lower and upper whiskers are 5 and 95th percentiles, respectively.

Further, significant metabolic features responsible for the separation between the groups were identified based on their variable influence on the projection (VIP) scores in the PLS-DA model. In general, significant metabolites were selected based on VIP scores of more ≥1; however, a stringent VIP score of 2 has been selected in the present study to screen out the metabolites of discriminatory significance. The metabolites ranked according to their VIP scores were identified using the metabolite assignment shown in Figure S4 and then labeled on the corresponding VIP score plots as shown in Figures S5, S6. The pair wise analysis between NC and MNU treatment, showed a clear differentiation indicating significant metabolic alterations in MNU group. Overall, we identified 16 metabolites significantly perturbed in the sera of MNU treated animals compared to NC. These markers metabolic entities along with their chemical shifts, variable importance on projection (VIP) score and *p*-value are listed in Table 4. Compared with control group, MNU treatment had a significant elevation of VLDL/LDL, PUFA, choline, isoleucine, leucine, valine, alanine, proline, tyrosine, phenylalanine, NAG, OAG whereas, they were having decreased levels of glucose, lactate, creatine, trimethylamine-N-oxide, and formate. These metabolic changes observed in MNU treated rats might be related to multiple tumor-related metabolic pathways, involving energy metabolism, amino acid metabolism, fatty acid metabolism and choline phospholipid metabolism. As evident from Table 4, these metabolic alterations were found to get ameliorated partially after the treatment, however, different treatments resulted in a different metabolic response (Table 4). The treatment effect is also pictorially depicted through representative box plots shown in Figure 8. To summarize, the zaltoprofen treatment was effective in resetting the elevated serum levels of proline, alanine, valine, lipid metabolites (VLDL/LDL, PUFA), NAG and decreased serum levels of formate, lactate, TMAO, and creatine. Zileuton was well in range to reset the elevated serum levels of isoleucine, leucine, valine, alanine, proline, phenylalanine, lipid metabolites (VLDL/LDL, PUFA), and decreased serum levels of formate, lactate, and creatine. All in all, the combination therapy improved the serum metabolic profiles of branched chain and aromatic amino acids along with choline metabolism.

**Table 4:** Metabolic variability's among the groups treated with MNU, Zaltoprofen and Zileuton when compared to toxic control.

**#**	**Metabolite**	**^1^H (ppm)**	**Control vs. toxic control**	**Zaltoprofen vs. toxic control**	**Zileuton vs. toxic control**	**Zaltoprofen + Zileuton vs. toxic control**
1	LDL/VLDL	0.89/1.27	↓↓↓↓	↓↓↓	↓↓↓	↑
2	Iso/Leucine	0.95	↓↓↓↓	↓↓	↓↓↓	↓↓↓
3	Valine	0.97	↓↓↓↓	↓↓↓	↓↓↓	↓↓↓
4	Lactate	1.31	↑↑↑↑	↑↑↑	↑↑	↑↑↑
5	Alanine	1.45	↓↓↓↓	↓↓	↓↓	↓↓↓
6	Proline	1.99	↓↓↓↓	↓↓↓	↓↓	↓
7	NAG	2.01	↓↓↓↓	↓↓↓	↓↓	↓↓
8	OAG	2.11	↓↓↓↓	↓↓↓	↑↑↑	↑↑↑
9	Creatine	3.01	↑↑↑↑	↑↑	↑↑↑	↑↑
10	Choline	3.19	↓↓↓↓	↑↑↑	↑↑	↓↓↓
11	TMAO	3.25	↑↑↑↑	–	↑↑↑	↑↑
12	Glucose	3.39	↑↑↑↑	↓↓↓	↓↓	↓
13	PUFA	5.31	↓↓↓↓	↓↓↓	↓↓	↑↑
14	Tyrosine	7.17	↓↓↓↓	↑↑	↓	–
15	Phenylalanine	7.31	↓↓↓↓	↓↓↓	↓↓	–
16	Formate	8.43	↑↑↑↑	↑#	↑#	↑#

*The up and down arrows represent, respectively, increased and decreased metabolite levels. A ↑↑↑/↓↓↓ or ↑↑/↓↓ score was given to the metabolites of the treatment dose which showed ameliorating effects from MNU toward control. Abbreviations used are as follows: LDL (low density lipoproteins); VLDL, very low density lipoproteins; NAG, N-acetyl glycoprotein; OAG, O-acetyl glycoprotein; PUFA, poly unsaturated fatty acids*.

*Symbols # = VIP Score < 1; –Level similar to control*.

## Discussion

The present study was inquested to elucidate the effect of DuCLOX-2/5 inhibition against MNU induced mammary gland carcinogenesis. Zaltoprofen (a specific COX-2 inhibitor) and zileuton (a specific 5-LOX inhibitor) were scrutinized alone and as a combination regime against MNU induced mammary gland carcinogenesis.

The precedent studies have suggested the participation of the reactive oxygen species (ROS) in conjunction with the AA mediated inflammatory pathway in carcinogenesis. Considering the same, the authors evaluated the oxidative stress biomarkers primarily relying on inflammation and carcinogenesis. In a more highlighted view, we scrutinized the enzymatic (SOD/catalase/GSH) and peroxidative biomarkers [TBARs and PC] in mammary gland tissue. We observed an increase in both the TBARs and PC in MNU treated group, which was restored back to normal after the treatments with drugs in question. Moreover, antioxidant enzyme's SOD, catalase, and GSH constitute the major supportive defense against free radicals and all work in tandem. SOD scavenges the superoxide radicals to form hydrogen peroxide (H_2_O_2_), which is further catalyzed to H_2_O and O_2_. We observed a decrease in SOD, catalase and GSH in MNU treated groups affirming ROS attack, and the same could be attributed to cell toxicity. Collectively, this can be stated that the proposed regime of DuCLOX-2/5 inhibition can impart favorable regulation of oxidative stress markers in MNU induced carcinogenesis.

In the emergence of the risk factors associated with the chemotherapeutic agents, autonomic dysfunction and associated cardiovascular abnormalities are among the most prevailing complications (Albini et al., 2010; Ewer and Ewer, 2010). In consideration to the cardinal damages and deterioration of the autonomic physiology, we also scrutinized the same using time and frequency domain in HRV parameters. It would be appropriate to mention that HRV is the most utilized non-invasive marker to study post chemotherapeutic autonomic dysfunction. In ECG profiling, MNU administration was found to be evident for prolongation of the QRS, QT, and QTc interval reflecting the cardiac risks with the carcinogenic. QRS complex indicates the ventricular depolarization, and the prolongation of the QT interval reflects the patients risk for the cardiac damage. Treatment with monotherapy and combination therapy of zaltoprofen and zileuton demarcated a significant effect toward restoration of QRS, QT, and QTc interval. The time and frequency-domain parameters for HRV were also favorably regulated by the proposed regime, implicating considerable regulation of autonomic control during cancer progression.

The whole mount preparations are often used as an appropriate method for the examination of small proliferative lesions as represented as an increase in the number of AB/TEBs and lobules; the corresponding structures in the human breast are the terminal ductal lobular units (Russo et al., 1990). Persistent TEBs implies the deregulation of apoptosis. The undifferentiated AB/TEBs are the sites for the malignant transformations and growth in AB/TEBs number is a direct indicator of proliferative breast tissue. The MNU treatment was evident as the increase in AB/TEBs count and DF score, which is in corroboration with the previous findings (Manral et al., 2016; Rani et al., 2016). Treatment with monotherapy and combination therapy significantly curtailed down the AB/TEB count and DF score. For validation of the morphology observed with the carmine staining, mammary gland tissues were further examined histopathologically. Mammary gland tissue from the control animals revealed the presence of duct, adipocytes, LCT, DCT, lymphocytes, CEC, and MEC. MNU treatment was recorded to have distorted cellular architecture, with scattered cuboidal epithelial cells and loss of adipocytes, which is in line with the previous reports and same, has been restored with treatment groups.

Previous reports endorsed the combined inhibition of COX-2 and 5-LOX leads to apoptotic signaling in the malignant cells via modulating balance between death and anti-death proteins (Schroeder et al., 2007). Therefore, we scrutinized the markers of mitochondrial apoptosis to validate the efficacy of DuCLOX-2/5 inhibition. Test groups alone and combination group decreased anti-apoptotic BCL-2 and BCL-xl protein levels. In a similar line, the expression of pro-apoptotic proteins BAD and BAX was found to be diminished in MNU toxic group and again normalized in the test groups. The same was justified significantly with the RT-PCR when contemplated against genomic expression of proteins of apoptosis. The monotherapy and combination therapy showed the significant impact on expression of VDAC, cytochrome-c, Apaf-1, and procaspase-9 toward normal. In mitochondrial apoptosis pathway, cytochrome-c (acts as an intracellular death signal) combines with a cytosolic protein called Apaf-1 (apoptotic protein activating factor-1) to form a complex called the apoptosome. Apaf-1 is a scaffold chaperon like protein which when activated by cytochrome-c recruits and activates procaspase-9 (Bratton and Salvesen, 2010). On activation of procaspase-9 to caspase-9, it activates downstream effector caspase-3/7, and promotes apoptosis (Ghosh and Karin, 2002; McIlwain et al., 2013). However, Benedict et al. performed the analysis of normal tissue mRNAs to examine the relative expression of the Apaf-1 isoforms in activation of procaspase-9. Experiment demonstrated the expression of multiple Apaf-1 isoforms in cancer cells, and specific isoform activate procaspase- 9 in response to cytochrome-c and dATP, and form apoptosomes (Benedict et al., 2000). Hence, one can commensurate that the increase in free APAF-1 expression, despite the overexpression of cytochrome-c, in MNU treated experimental groups directly reflect its unbound form present in cells. The same could be justified while conferring levels of procaspase-9 in toxic treatment. Apaf-1 and procaspase-9 were found to be overexpressed reflecting its inactivated forms, which are unable to form a complex to induce apoptosis after MNU treatment.

Considering the findings from the preceding paragraph, authors find it more justifiable to extend the dimension of evidence through genomic expression for the observed phenotype. The genomic contributors' expression through quantitative RT-PCR mirrors the immunoblotting studies, and confirms the effective treatment of DuCLOX inhibitor.

The current study also established the apoptotic potential of zaltoprofen and zileuton when evaluated for caspase activity biochemically. Subsequential significant upsurge of caspase-8 and caspase-3 levels were found in test compounds, alone or in combination, against MNU administration (Cheung et al., 2006). In death inducing signaling pathway, caspase-8 from the extrinsic pathway directly activates caspase-3, and facilitates the release of cytochrome-c, which both proteins are imperative part of the intrinsic pathway (Zhuang et al., 1999; Steward and Brown, 2013). From above, it became conspicuous that DuCLOX-2/5 inhibition treatment curtailed down the proliferative and anti-apoptotic effects of MNU when affirmed through the mitochondrial mediated apoptotic pathway.

To make the study more defensible for the role of DuCLOX-2/5 inhibition in cancer progression, the COX-2 and 5-LOX gene expression were assessed along with the mitochondrial apoptotic proteins. Since, the test drugs were well reported to be specific inhibitors of COX-2 and 5-LOX, RT-PCR and immunoblotting confirmed the expression of these enzymes concomitantly curtailed down with the test drug treatment.

NMR-based serum metabolomics in conjunction with multivariate data analysis revealed the metabolic profile of MNU treated rats, and those treated with dose zaltoprofen and zileuton alone, and in combination compared to toxic control. The treatment groups D1, D2 and D3 were shifted relatively close toward control, showing the improving effects from the treatment. Based on the metabolic perturbation in toxic control with respect to that of control (Table 4) ameliorating effects of zaltoprofen and zileuton alone and in combination were assessed. We observed a significant increase in the levels of PUFAs, lipoproteins (LDL/VLDL), and choline in the TC rats compared to NC, suggesting altered phospholipid metabolism (choline/GPC) and fatty acid metabolism (PUFAs, LDL/VLDL) in TC rats. The lipoproteins (VLDL/LDL) mainly transports cholesterols, oxysterols and triglycerides from the liver to rapidly proliferating cancer cells where it is used in membrane biogenesis, protein modifications, and steroid hormone production (Flote et al., 2016). Further fatty acids and lipids are also consumed through β-oxidation to meet the energy requirement for cell membrane synthesis, rapid proliferation and cancer cell survival (Zhang and Du, 2012). On the contrary, PUFAs and choline are important intermediates of membrane metabolism and inflammatory mediators (Raphael and Sordillo, 2013). Therefore, the elevated levels might be related to their augmented utilization to repair the damaged cells and dampen the inflammation associated with TC induced injury to mammary gland cells (Zhang and Spite, 2012). Chronic inflammation is a common clinical manifestation of various cancer types. Consistent with this the increased levels of N and O -acetyl glycoproteins (NAG and OAG) were present in the rat sera. The NAG and OAG are acute phase proteins and are expressed more during infection, trauma, surgery, and inflammation (Saldova et al., 2007) and are consistent with various types of cancers (Jobard et al., 2014). Both cancer and inflammation are known to trigger a hyper-catabolic state, resulting in increased energy requirements and protein metabolism. Consistent with this phenomenon, the decreased serum levels of glucose and lactate indicate the increased energy demand in these rats to sustain the active inflammatory processes and cell proliferation activities. The decreased levels of serum glucose in the animals imply that the glucose is being rapidly consumed by aerobic glycolysis for tumor cell proliferation and growth, which is consistent with the “Warburg effect” (Vander Heiden et al., 2009). The significant increase in serum lactate levels has been instituted in many cancer's studies. Nevertheless, in our study the serum lactate levels were found to be depleted, as cancer cells take up the lactate and use it to feed cancer cell mitochondrial energy production and to generate mitochondrial precursors for cancer cell biogenesis also called as “reverse Warburg's effect” (Wallace, 2012). To maintain physiological homeostasis and meet the energy requirements of cancerous cells, there is an increased reliance on alternate energy substrates preferably amino acids. Amino acids serve as a major source of energy, especially during conditions in which glucose availability is limited. Muscles along with the liver release high quantity of amino acids present in the body to maintain the cellular homeostasis in conditions of energy deprivation (Schutz, 2011). Consistent with this, increased levels of several amino acids in the sera such as alanine, proline, branched chain amino acid (isoleucine, leucine, valine-BCAA) and aromatic amino acid (phenylalanine, tyrosine-AAA) suggests aberrant amino acid metabolism. The amino acids are broken down into pyruvate, alpha-ketoglutarate, succinyl-CoA, fumarate, and/or oxaloacetate that can be predominantly converted into glucose or glycogen via TCA cycle or gluconeogenesis to generate energy during stress (DeBerardinis et al., 2008). The decreased level of creatine is supposed to compensate for the lower efficiency of ATP production and act as an alternate energy source for cancer proliferation. TMAO is a product of gut microflora activity; decreased levels might be the consequence of adaptation to the disease state (Hartiala et al., 2016). Formate is primarily derived from mitochondrial metabolism and is the precursor molecule required to make DNA and other critical compounds within the cell (Ahn and Metallo, 2015; Newman and Maddocks, 2017) and might be responsible for their decreased level in the serum. As evident from our study the up and down regulated metabolites, suggest perturbed glycolysis, beta-oxidation pathway, and deranged mitochondrial activity. Henceforth, authors would like to submit that zaltoprofen, zileuton and a combination dose can favorably regulate the metabolic alterations induced by MNU.

**Figure 9:**
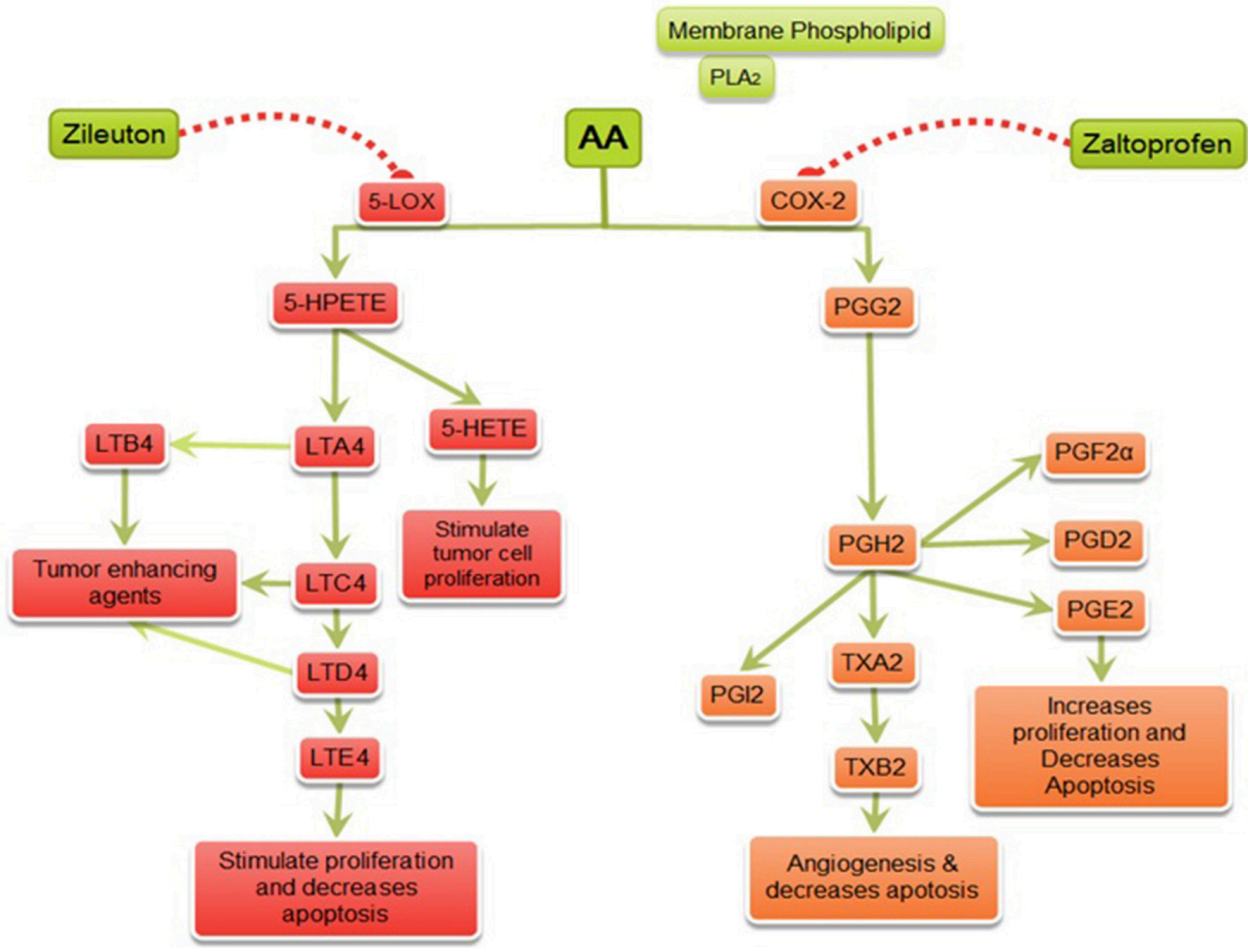
Mechanism of action of zaltoprofen and zileuton. PLA_2_, Phospholipase A_2_; AA, Arachidonic acid; 5-LOX, 5-Lipoxygenase; 5-HETE, 5-hydroxytetraenoic acid; LTA_4_, leukotriene A_4_; LTB_4_, leukotriene B_4_; LTC_4_, leukotriene C_4_; LTD_4_, leukotriene D_4_; LTE_4_, leukotriene E_4_; PGE2, prostaglandin E_2_; PGF2α, Prostaglandin F2α; PGD2, prostaglandin D_2;_ PGI_2_, prostacyclin; PGH_2_, prostaglandin H_2;_ TXA_2_, Thromboxane A_2_; TXB_2_, Thromboxane B_2_; {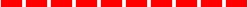 denotes inhibition; 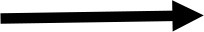 denotes activation}.

In summary, this study identified DuCLOX-2/5 inhibition as chemopreventive targets of mammary gland cancer. Their specific inhibitors prevented MNU-induced mammary gland carcinogenesis through their inhibitory effects on AA metabolism (Figure 9). Considering the lack of pharmaceutical agents with the potential to inhibit DuCLOX-2/5 in particular, we believe further studies are needed to be implemented in a safe and efficacious strategy for prevention of human mammary gland cancer.

## Author contributions

SG carried out the bench work; AR performed the NMR metabolic studies; SS performed the qRT-PCR studies; SR, MS, UD, and RY performed the carmine staining and histopathology; LS and JR performed the caspase3 and caspase8 assay; MA and AS performed the statistical studies and compiled the data; DK evaluated the results of NMR metabolic studies; RP supervised the qRT-PCR studies; GK perceived the idea, designed and supervised the whole study, prepared and proof read the final manuscript.

### Conflict of interest statement

The authors declare that the research was conducted in the absence of any commercial or financial relationships that could be construed as a potential conflict of interest.

## Supplementary material

Figure S1 ECG recording. Group I: Control (Normal saline, 3 ml/kg, p.o.), Group II: Toxic control (47 mg/kg MNU, i.v.), Group III: Zaltoprofen (10 mg/kg, p.o. + MNU 47 mg/kg, i.v.), Group IV: Zileuton (10 mg/kg, p.o. + MNU 47 mg/kg, i.v.), Group V: Zaltoprofen + Zileuton (5 +5 mg/kg, p.o. + MNU 47 mg/kg, i.v.).

Figure S2 The ECG and HRV markers identified from PLS-DA analysis and are listed in decreasing order of VIP score to highlight their discriminatory potential. In **(A,B)**, the complete HRV was used to PLS-DA modeling and resulted VIP scores for time and frequency domain are shown. Group 1: Control (Normal saline, 3 ml/kg, p.o.), Group 2: Toxic control (47 mg/kg MNU, i.v.), Group 3: Zaltoprofen (10 mg/kg, p.o. + MNU 47 mg/kg, i.v.), Group IV: Zileuton (10 mg/kg, p.o. + MNU 47 mg/kg, i.v.), Group V: Zaltoprofen + Zileuton (5 +5 mg/kg, p.o. + MNU 47 mg/kg, i.v.).

Figure S3 The 2D PCA score plots for pairwise analysis. The pair wise 2D PCA, PLS-DA, and OPLS-DA score plots with their respective R^2^ and Q^2^ values derived from 1D ^1^H CPMG NMR spectra. Groups were differentiated as: NC- Normal control (Normal saline, 3 ml/kg, p.o.), TC-Toxic control (MNU 47 mg/kg, i.v.), D1- Zaltoprofen (10 mg/kg, p.o. + MNU 47 mg/kg, i.v.), D2- Zileuton (10 mg/kg, p.o. + MNU 47 mg/kg, i.v.) and D3-Zaltoprofen+Zileuton-(5 +5 mg/kg, p.o. + MNU 47 mg/kg, i.v.). **(A)** NC vs. TC, **(B)** NC vs. D1, **(C)** NC vs. D2, **(D)** NC vs. D3. Colored circles indicate the 95% confidence interval for each class.

Figure S4 Stack plot of representative 1D ^1^H NMR spectra of rat sera obtained from different groups. The representative1D ^1^H CPMG NMR spectra of rat serum obtained from different groups. The peaks annotated in the Figure show the assignments of serum metabolites. Groups were differentiated as: NC- Normal control (Normal saline, 3 ml/kg, p.o.), TC-Toxic control (MNU 47 mg/kg, i.v.), D1- Zaltoprofen (10 mg/kg, p.o. + MNU 47 mg/kg, i.v.), D2- Zileuton (10 mg/kg, p.o. + MNU 47 mg/kg, i.v.) and D3-Zaltoprofen+Zileuton-(5 + 5 mg/kg, p.o. + MNU 47 mg/kg, i.v.). The abbreviations used are: LDL/VLDL, Low/very-low density lipoproteins; PUFA, polyunsaturated fatty acids; BCAA, Isoleucine, Leucine, Valine; 3-HB, 3-hydroxy-butyrate; Ala, Alanine; Arg, Arginine; Lys, Lysine; NAG, N-acetyl glycoproteins; OAG, O-acetyl glycoprotein; Pro, Proline; Glu, Glutamate; Gln, Glutamine; TMAO, Trimethylamine-N-oxide; Gly, Glycine; Thr, Threonine.

Figure S5 The potential biomarker metabolite entities identified from PLS-DA analysis and are listed in decreasing order of VIP score to highlight their discriminatory potential. In **(A)**, the complete NMR data matrix was used to PLS-DA modeling and resulted VIP scores for top 35 metabolite entities are shown. In **(B)**, the down-field spectral region from 5.4 to 9.5 ppm was used for PLS-DA modeling and revealed the discriminatory importance of aromatic amino acids like Histidine, Tyrosine and phenylalanine. In **(C)**, the up-field spectral region from 0.9 to 4.5 ppm was used for PLS-DA modeling and revealed the discriminatory importance of other serum metabolites mainly amino acids and metabolites of tricarboxylic acid cycle. Groups were differentiated as: NC- Normal control (Normal saline, 3 ml/kg, p.o.), TC-Toxic control (MNU 47 mg/kg, i.v.), D1- Zaltoprofen (10 mg/kg, p.o. + MNU 47 mg/kg, i.v.), D2- Zileuton (10 mg/kg, p.o. + MNU 47 mg/kg, i.v.) and D3-Zaltoprofen+Zileuton-(5 + 5 mg/kg, p.o. + MNU 47 mg/kg, i.v.).

Figure S6 Same as Figure S5.

## Abbreviations

AA: arachidonic acid
AB: alveolar bud
BSA: bovine serum albumin
CEC: cuboidal epithelial cell
COX-2: Cyclooxygenase-2
DHA: docosahexaenoic acid
DCT: dense connective tissue
ECG: electrocardiogram
GSH: glutathione
GPC: glycerophosphocholine
H&E: hematoxylin & eosin
HRV: heart rate variability
HR: heart rate
HMBD: the human metabolome database
HF: high frequency
LF: low frequency
LCT: loose connective tissue
5-LOX: 5-lipoxygenase
LDL: low density lipoprotein
MEC: myoepithelial cell
MNU: Methyl Nitrosourea
NAG: N-acetyl glycoprotein
OAG: O-acetyl glycoprotein
PUFA: polyunsaturated fatty acid
PS: phosphatidylserine
PC: phosphocholine
PCA: principal component analysis
PLS-DA: partial least squares discriminant analysis
ROS: reactive oxygen species
SOD: superoxide dismutase
TBARs: thiobarbituric acid reactive substances
TSP: trimethylsilylpropionic acid-d4
VLF: very low frequency
VLDL: very low density lipoprotein.
