# Supplementary figures and images for: DuCLOX-2/5 Inhibition Attenuates Inflammatory Response and Induces Mitochondrial Apoptosis for Mammary Gland Chemoprevention
Source: Front Pharmacol. 2018 Apr 6;9:314. doi: 10.3389/fphar.2018.00314 (PMC5897656; doi:10.3389/fphar.2018.00314)

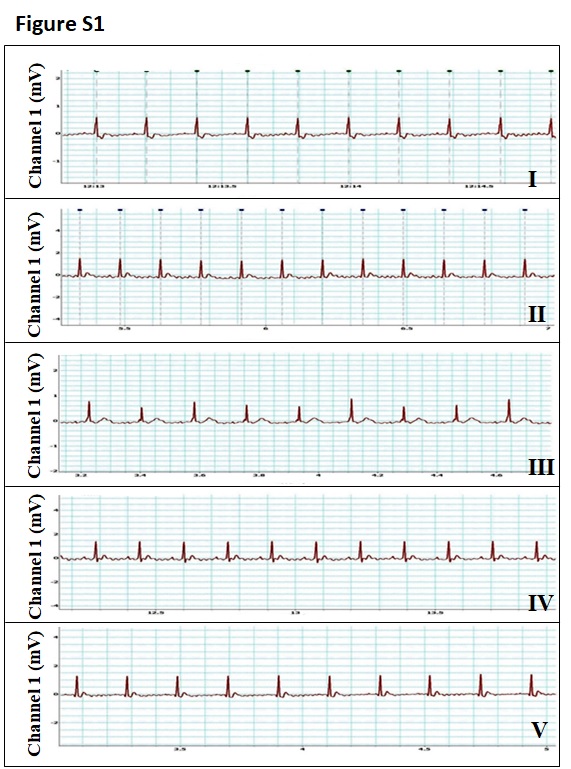

Supplement: Figure S1 — ECG recording. Group I: Control (Normal saline, 3 ml/kg, p.o.), Group II: Toxic control (47 mg/kg MNU, i.v.), Group III: Zaltoprofen (10 mg/kg, p.o. + MNU 47 mg/kg, i.v.), Group IV: Zileuton (10 mg/kg, p.o. + MNU 47 mg/kg, i.v.), Group V: Zaltoprofen + Zileuton (5 +5 mg/kg, p.o. + MNU 47 mg/kg, i.v.). [file Image1.JPEG]

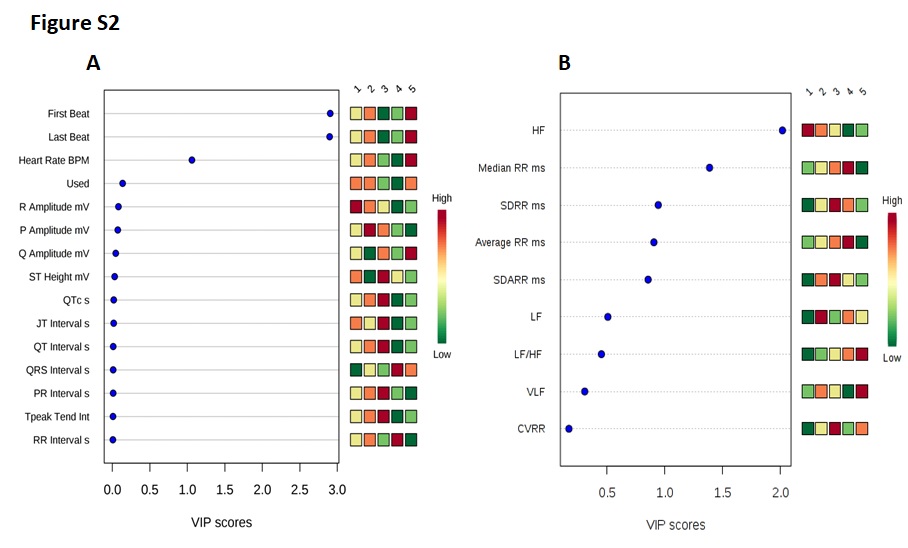

Supplement: Figure S2 — The ECG and HRV markers identified from PLS-DA analysis and are listed in decreasing order of VIP score to highlight their discriminatory potential. In (A,B), the complete HRV was used to PLS-DA modeling and resulted VIP scores for time and frequency domain are shown. Group 1: Control (Normal saline, 3 ml/kg, p.o.), Group 2: Toxic control (47 mg/kg MNU, i.v.), Group 3: Zaltoprofen (10 mg/kg, p.o. + MNU 47 mg/kg, i.v.), Group IV: Zileuton (10 mg/kg, p.o. + MNU 47 mg/kg, i.v.), Group V: Zaltoprofen + Zileuton (5 +5 mg/kg, p.o. + MNU 47 mg/kg, i.v.). [file Image2.JPEG]

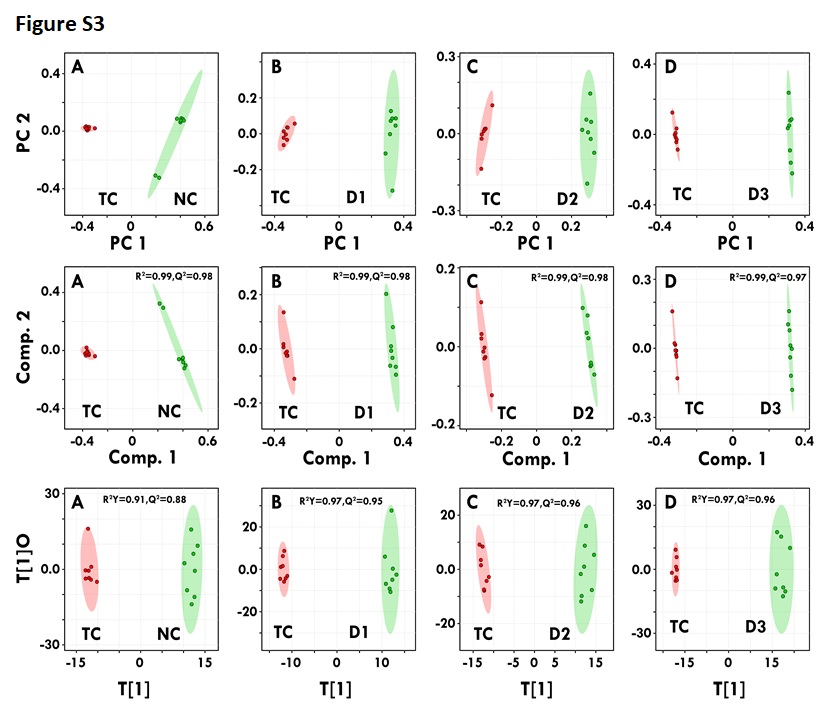

Supplement: Figure S3 — The 2D PCA score plots for pairwise analysis. The pair wise 2D PCA, PLS-DA, and OPLS-DA score plots with their respective R2 and Q2 values derived from 1D 1H CPMG NMR spectra. Groups were differentiated as: NC- Normal control (Normal saline, 3 ml/kg, p.o.), TC-Toxic control (MNU 47 mg/kg, i.v.), D1- Zaltoprofen (10 mg/kg, p.o. + MNU 47 mg/kg, i.v.), D2- Zileuton (10 mg/kg, p.o. + MNU 47 mg/kg, i.v.) and D3-Zaltoprofen+Zileuton-(5 +5 mg/kg, p.o. + MNU 47 mg/kg, i.v.). (A) NC vs. TC, (B) NC vs. D1, (C) NC vs. D2, (D) NC vs. D3. Colored circles indicate the 95% confidence interval for each class. [file Image3.JPEG]

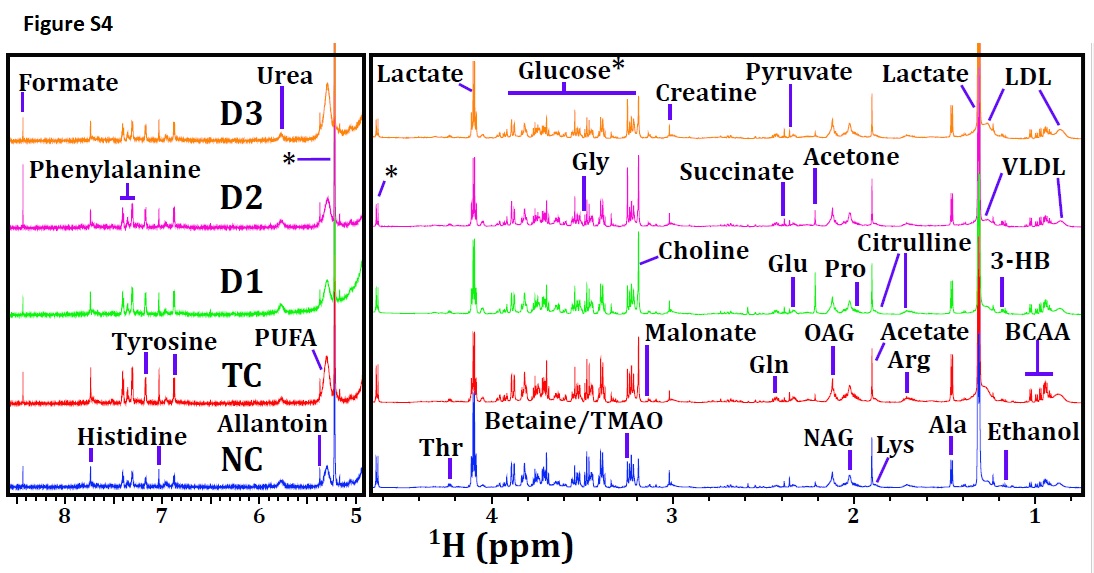

Supplement: Figure S4 — Stack plot of representative 1D 1H NMR spectra of rat sera obtained from different groups. The representative1D 1H CPMG NMR spectra of rat serum obtained from different groups. The peaks annotated in the Figure show the assignments of serum metabolites. Groups were differentiated as: NC- Normal control (Normal saline, 3 ml/kg, p.o.), TC-Toxic control (MNU 47 mg/kg, i.v.), D1- Zaltoprofen (10 mg/kg, p.o. + MNU 47 mg/kg, i.v.), D2- Zileuton (10 mg/kg, p.o. + MNU 47 mg/kg, i.v.) and D3-Zaltoprofen+Zileuton-(5 + 5 mg/kg, p.o. + MNU 47 mg/kg, i.v.). The abbreviations used are: LDL/VLDL, Low/very-low density lipoproteins; PUFA, polyunsaturated fatty acids; BCAA, Isoleucine, Leucine, Valine; 3-HB, 3-hydroxy-butyrate; Ala, Alanine; Arg, Arginine; Lys, Lysine; NAG, N-acetyl glycoproteins; OAG, O-acetyl glycoprotein; Pro, Proline; Glu, Glutamate; Gln, Glutamine; TMAO, Trimethylamine-N-oxide; Gly, Glycine; Thr, Threonine. [file Image4.JPEG]

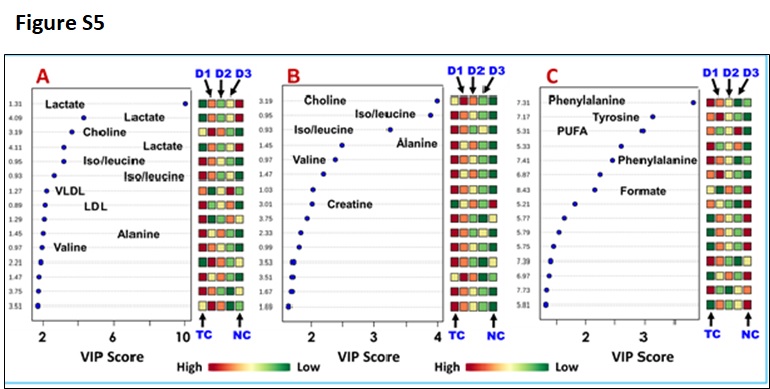

Supplement: Figure S5 — The potential biomarker metabolite entities identified from PLS-DA analysis and are listed in decreasing order of VIP score to highlight their discriminatory potential. In (A), the complete NMR data matrix was used to PLS-DA modeling and resulted VIP scores for top 35 metabolite entities are shown. In (B), the down-field spectral region from 5.4 to 9.5 ppm was used for PLS-DA modeling and revealed the discriminatory importance of aromatic amino acids like Histidine, Tyrosine and phenylalanine. In (C), the up-field spectral region from 0.9 to 4.5 ppm was used for PLS-DA modeling and revealed the discriminatory importance of other serum metabolites mainly amino acids and metabolites of tricarboxylic acid cycle. Groups were differentiated as: NC- Normal control (Normal saline, 3 ml/kg, p.o.), TC-Toxic control (MNU 47 mg/kg, i.v.), D1- Zaltoprofen (10 mg/kg, p.o. + MNU 47 mg/kg, i.v.), D2- Zileuton (10 mg/kg, p.o. + MNU 47 mg/kg, i.v.) and D3-Zaltoprofen+Zileuton-(5 + 5 mg/kg, p.o. + MNU 47 mg/kg, i.v.). [file Image5.JPEG]

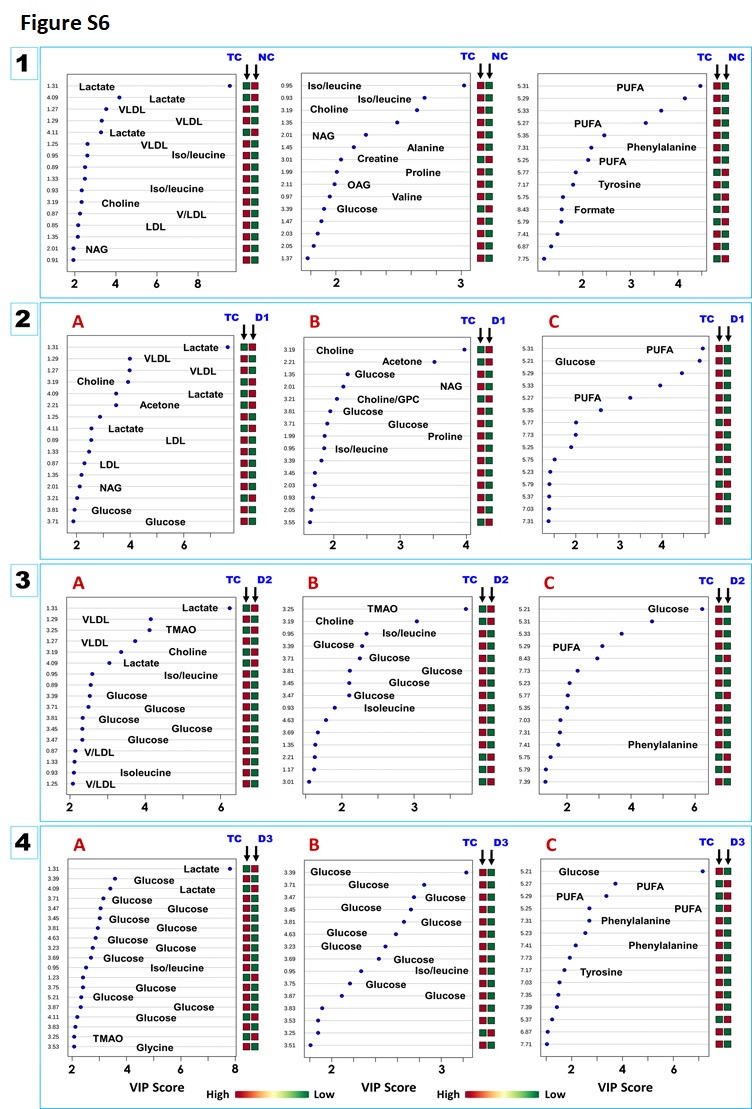

Supplement: Figure S6 — Same as Figure S5. [file Image6.JPEG]
